# Organo-Functionalization: An Effective Method in Enhancing the Separation and Antifouling Performance of Thin-Film Nanocomposite Membranes by Improving the Uniform Dispersion of Palygorskite Nanoparticles

**DOI:** 10.3390/membranes11110889

**Published:** 2021-11-19

**Authors:** Liu Yang, Qianwen Zhang, Qikun Wang, Wande Ding, Kefeng Zhang

**Affiliations:** 1 School of Municipal and Environmental Engineering, Shandong Jianzhu University, Jinan 250101, China; 15665888192@163.com (L.Y.); qikunwang12@gmail.com (Q.W.); kfz@sdjzu.edu.cn (K.Z.); 2 School of Water Resources & Environment, China University of Geosciences, Beijing 100083, China; zhangqw@caep.org.cn; 3 Shandong Shuifa Environmental Technology Co., Ltd., Jining 272000, China

**Keywords:** reverse osmosis, palygorskite nanoparticles, organo-functionalization, desalination, antifouling

## Abstract

Recently, palygorskite (Pal) has become a promising new membrane additive in flux enhancement and fouling reduction, which is an environmentally friendly nanoclay material under the 2:1 layer composition with 1D tubular structure. However, the aggregation of Pal due to the intermolecular forces is still an obstacle to be solved in improving membrane performance. Herein, Pal nanoparticles were chemically modified by KH550 to weaken the aggregation and improve the dispersibility, and then incorporated into the organic phase to prepare thin-film nanocomposite (TFN) membranes. The results showed that the organo-functionalization could effectively improve the membrane hydrophilicity and dispersion of Pal nanoparticles in the polyamide layer, which contributed to the enhanced water flux (from 25 to 38 L/m^2^·h), unchanged salt rejection (98.0%) and better antifouling capacity (91% flux recovery rate), which suggested that the organo-functionalization of nanoparticles was an efficient method in further enhancing membrane performance

## 1. Introduction

Recently, clay nanoparticles have been popular since they are naturally abundant and cost effective; in particular, the two-dimensional sheet structures and inherent hydrophilicity make them potential ideal nanofillers for thin-film nanocomposite (TFN) membranes [1,2]. Clay materials, such as halloysite nanotubes (HNTs), layered double hydroxides (LDHs) and montmorillonites (MMTs), have been added into mixed matrix membranes and presented the positive effect on the TFN membrane performance, not only the enhanced water flux, but also improved antifouling capacity and chlorine resistance [3,4,5]. Except for the clays mentioned above, palygorskite (Pal) is another special class of clay mineral under the 2:1 layer composition with 1D rod-like morphology [6]. The nanoscale porous structure with the cross-sectional area of 0.37 nm × 0.63 nm could provide extra parallel nanochannels in the polyamide (PA) layer and make it conducive to the transport of the water molecules, which has drawn more attention as capable membrane nanofillers [7,8].

Ji et al. introduced the raw Pal nanoclay in the PVDF ultrafiltration flat sheet membranes, and the pure water flux showed a sharp increase from 106 to 221 L/m^2^·h [9]. Wei et al. synthesized novel ultrafiltration membranes by incorporating Pal into a polyvinylidene fluoride (PVDF) matrix. The water flux and flux recovery rate (FRR) under bovine serum albumin (BSA) pollution of the composite membranes were all superior to those of the pristine membranes [10]. By a vacuum-assisted filtration self-assembly process, Zhao et al. fabricated free-standing GOP membranes based on GO nanosheets and Pal nanorods. The permeate fluxes increased by seven times for GOP membrane with excellent separation performance and anti-oil-fouling properties during oil-in-water emulsion separation test [11]. In addition, TiO_2_/Pal and silver/Pal nanoparticles were synthesized by Wang et al., and then were incorporated in the TFN membrane separating layer through interfacial polymerization [2,6]. The results showed that both TiO_2_/Pal and Ag/Pal nanoparticles brought the excellent photocatalytic bactericidal and antifouling capacities of the TFN membranes.

However, despite the significant achievements in Pal-based membranes, it can not be ignored that similar to other nanoparticles, the aggregation of Pal also easily occurred during the membrane fabrication due to the intermolecular forces, which have a negative influence on the membrane separation performance [12]. Thus, it is essential to weaken the aggregation of Pal in order to improve the dispersion and avoid introducing a lot of defects in the membrane. Till now, many attempts have been made, which can be summarized as the following four processes: (1) acidified processing, (2) surfactant processing, (3) coupling agent surface treatment and (4) ultrasonic wave processing [12]. Among them, 3-aminopropyltriethoxy silane coupling agent (KH550) was widely used in the Pal modification due to its special structure, which has been proved to be an effective surface functionalization method to improve the dispersibility of the nanoparticles. The coupling process can be accomplished via the chemical reaction between the triethoxy groups of silane molecules and the hydroxyl groups on the Pal surface, whereas the other functional group of silane molecule can remain [13]. Zhang et al. modified the Pal nanoparticles by KH550, and then incorporated into the PVDF matrix to develop a hybrid membrane via a phase inversion method [12]. The results showed that the saline coupling agent KH550 achieved a more uniform presence of Pal in the polymer matrix than that of the raw Pal, and the membrane exhibited better hydrophilicity, thermal stability, permeation and antifouling properties. In addition, Han et al. modified Pal by KH550 to improve the dispersion and water loss rate of zeolite in asphalt, and the result showed that KH550-Zeolite had better compatibility and could stay longer in n-heptane [14].

Above all, the modification of Pal by KH550 seems to be an effective method to improve the dispersibility of the nanoparticles. However, up to now, most of the reported literature put emphasis on the MF and UF membrane performance based on the addition of KH550-modified Pal; there are rare studies focusing on the effect of the modification of Pal by KH550 on the structure, separation and antifouling performance of TFN membranes. Besides, the effect of particle size and loading content of Pal on the water flux and salt rejection of TFN membranes has also not been systematically and comprehensively studied. These motivated our work and was believed to pave a new avenue in enhancing the dispersion of other nanoparticles in the fabrication of the TFN membranes.

**Figure 1:**
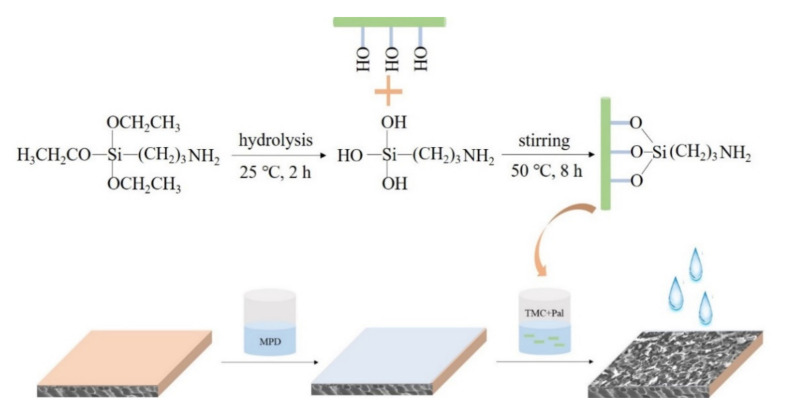
Chemical modification mechanism of Pal and membrane fabrication procedure.

Therefore, in the current work, Pal nanoparticles were used as additives in the organic phase to improve the performance of TFN membranes. The effects of particle size, loading content and KH550 modification of Pal on the water flux, salt rejection, stability and antifouling capacity of the prepared TFN membranes were evaluated. Besides, several characterization methods such as FTIR, XRD, SEM and TEM were undertaken in order to confirm the structural properties of both Pal nanoparticles and RO membranes. The chemical modification mechanism of Pal and membrane fabrication procedure were shown in Figure 1.

## 2. Experimental Section

### 2.1. Materials and Chemicals 

Polysulfone (PS) ultrafiltration membrane was supplied by Beijng Originwater Technology Co., Ltd. (Beijing, China). Palygorskite (Pal) was provided from Jiangsu Shengyi Nano Technology Co., Ltd. (Jiangsu, China). γ-aminopropyl triethoxysilane (KH550), humic acid (HA), Trimesoyl chloride (TMC, >98%) and m-phenylenediamine (MPD, >99%) were all purchased from Shanghai Aladdin Chemistry Co., Ltd. (Shanghai, China). Ethyl alcohol, sodium dodecyl sulfate (SDS), n-hexane, sodium chloride (NaCl) and magnesium chloride (MgCl_2_) were obtained from Sinopharm Chemical Reagent Co., Ltd. (Shanghai, China). All the solutions were prepared using deionized (DI) water as solvent for RO measurements.

### 2.2. Preparation of the Modified Pal Nanoparticles

To achieve shorter Pal nanoparticles in diameter, facile grinding technology was applied [15,16]. In brief, a certain amount of raw Pal was put in miniature omnidirectional planetary ball mill (Hunan Focucy experimental instrument Co., Ltd., Hunan, China) for 60 min at 60 rpm. Spherical zirconia balls with a diameter of about 5.0 mm were used as the milling medium. To obtain the KH550-modified Pal, the KH550 was first hydrolyzed at 25 °C for 2 h using 200 mL ethanol and water as solvent (70% ethanol + 30% deionized water). Then, 5 g of grinded Pal was introduced and the mixture was stirred for another 8 h under N_2_ atmosphere at 50 °C. The mass ratio of KH550 and grinded Pal m/m was 0, 1:4, 3:4, 3:2, 3:1. The resulting salivated material was consecutively rinsed with ethanol, dried at 120 °C under vacuum and stored in the desiccators for next analysis. The raw Pal was abbreviated as Pal, the grinded Pal was abbreviated as g-Pal and the KH550-modified Pal was abbreviated as K-Pal.

### 2.3. Fabrication of RO Membranes

By using an interfacial polymerization (IP) method, TFC and TFN membranes were prepared [17]. Briefly, the substrate top surface was immersed in the 2 wt% MPD aqueous solution containing 0.1 wt% SDS for 2 min, in which the pH of the solution had been adjusted to 8.5. After removing the excessive MPD solution and dried by the nitrogen gas, 0.1 *w*/*v*% TMC in n-hexane solution containing various concentration of three types of Pal nanoparticles, from 0.00 to 0.07 wt% contacted with the MPD immersed membrane surface for 1 min. Then, the excess organic solution was removed, and the resultant membrane was oven-dried for 5 min at 80 °C, followed by washing thoroughly with DI water. It is noticed that ultra-sonication for at least 0.5 h was necessary when Pal nanoparticles were added in TMC solution, so as to make Pal nanoparticles well dispersed.

Herein, four kinds of Pal-based membrane were fabricated including pristine TFC membrane, TFN membrane with Pal nanoparticles, TFN membrane with g-Pal nanoparticles (smaller size) and TFN membranes with K-Pal nanoparticles. To distinguish different TFN membranes, the membranes with Pal nanoparticles, g-Pal nanoparticles (smaller size) and K-Pal nanoparticles were denoted as TFN-P_x_, TFN-G_x_ and TFN-K_x_ (x was the concentration (wt%) of Pal nanoparticles in the PA layer).

### 2.4. Characterization of Pal Nanoparticles and Membranes

#### 2.4.1. Transmission Electron Microscope (TEM)

Under ultrasonic wave (100 W), different Pal nanoparticles were dispersed in n-hexane solution. The suspended nanoparticles were dropped on a copper film and then were dried in air. The nanostructure of the particles was observed by using TEM (JEM-2100, JEOL, Tokyo, Japan) working at 200 kV.

#### 2.4.2. Dynamic Light Scattering (DLS)

To measure the average size of different Pal nanoparticles, the nanoparticles were dispersed in n-hexane solution with the aid of ultrasonic wave (100 W). Then, a certain amount of particle solution was transformed to the sample cell and analyzed by DLS (Malvern Zetasizer Nano series, Shanghai, China). The measurement wavelength was 635 nm and the detection angle was 90°.

To measure the Zeta potential of different Pal nanoparticles, the nanoparticles were dispersed in water solution with the aid of ultrasonic wave (100 W). Then, a certain amount of particle solution was transformed to the sample cell and analyzed by DLS under zeta potential measurement mode. The dispersant was water and the pH of particle solution ranged from 6.86 to 7.06.

#### 2.4.3. Fourier Transforms Infrared (FTIR)

The surface functional groups of different Pal nanoparticles and membranes were determined by using FTIR (Nicolet iS50). For Pal nanoparticles, the measurement was under TR testing mode; for membranes, the measurement was under ATR testing mode. Each spectrum was recorded at a resolution of 4 cm^−1^ in the range of 600–4000 cm^−1^.

#### 2.4.4. X-ray Diffraction (XRD)

To confirm the effect of grinding and chemical modification on the crystal structure of Pal nanoparticles, XRD (RigakuD/Max 2200PC) with CuKa radiation (λ = 0.15418 nm) at room temperature with the applied tube voltage and electric current at 40 kV and 20 mA was used. The 2θ was ranging from 5° to 90°.

#### 2.4.5. Contact Angle Measurement

Water contact angle was measured by an automatic contact angle meter (DSA100, Kruss, Shanghai, China). A 2 μL drop of distilled water was deposited on the sample surface using a syringe. When the water drop showed no further change or changed little, the drop image was registered by a video camera and image analysis software was used to calculate the contact angle. Each sample was repeated at least three times, so as to improve the accuracy of the contact angle results. For particle samples, it was measured after being pressed into thin sheets.

#### 2.4.6. Settling Analysis

The dispersion of Pal, g-Pal and K-Pal in TMC solution was evaluated to obtain the most suitable modification concentration by settling analysis. 0.03 g Pal, g-Pal and K-Pal nanoparticles were added in 250 mL *n*-hexane solution, respectively. The mixture was ultrasonic for 1 h and then transferred to the measuring cylinder for sedimentation observation. The sedimentation height was recorded every 4 h and lasted for 24 h.

#### 2.4.7. Scanning Electron Microscope (SEM)

To observe the surface morphology of different Pal nanoparticles and membranes, SEM (S-4800, Hitachi, Tokyo, Japan) was applied. All the particles and membranes were oven-dried overnight prior to analysis. For membranes, dry membrane samples were frozen in liquid nitrogen and subsequently cracked in order to obtain the cross sections. All the samples were sputter-coated with gold for 50 s and viewed at 10 kV.

#### 2.4.8. Atomic Force Microscopy (AFM)

By using in situ AFM (Veeco, Plainview, NY, USA), the membrane roughness was determined under tapping mode in air. The scanning area was 5 μm × 5 μm and Z-scale was 500 nm. The values of root-mean-squared height (RMS) reflect the magnitude of the surface roughness.

### 2.5. RO Performance 

Permeability and selectivity of TFC and TFN membranes were measured through cross-flow permeation test by using RO performance evaluation equipment with three 24 cm^2^ parallel filtration cells. NaCl and MgCl_2_ with a concentration about 2 g/L were used as the feed solution and the operation pressure was 1.6 MPa after compaction at 1.8 MPa for 0.5 h. Conductivity meter was applied to measure the NaCl and MgCl_2_ concentrations. The water flux (*J*, L/m^2^·h), water permeability (*A*, L/m^2^·h·bar) and salt rejection (*R*, %) of the prepared membranes were calculated with the following equations, respectively [18]:

(1) $$J=\frac{\Delta V}{S\cdot \Delta t}$$

(2) $$A=\frac{J}{\Delta P-\Delta \pi }$$

(3) $$R=1-\frac{C_{2}}{C_{1}}$$

where Δ*V* is the permeate volume, *S* is the effective membrane area, Δ*t* is the measuring time interval, Δ*P* is the transmembrane pressure, ∆π is the osmotic pressure of the feed solution and *C*_2_ and *C*_1_ are the permeate and feed concentration, respectively.

The stability of the membrane directly affects the membrane service life, thus the prepared TFC and TFN membranes were tested under different temperature, different operation pressure and long-term RO test using 2 g/L NaCl as feed solution, so as to determine the stability of the Pal-based TFN membranes.

### 2.6. Antifouling Ability

In order to determine the antifouling performance of the TFC and TFN membranes, HA was used as model foulant to conduct a fouling test. After the water flux reached stability, 0.5 g/L HA was added to the NaCl feed solution and continue the permeation test. The fouling filtration experiment lasted for 10 h, and then cleaned with DI water for 2 h. After that, the recovered water flux of the cleaned membrane was remeasured using a 2 g/L NaCl solution for 3 h. The flux recovery rate (*FRR*, %) was calculated by the following Equation [2]:

(4) $$FRR=\frac{J_{1}}{J_{0}}*100\%$$

where *J*_1_ is the recovered water flux after cleaning and *J*_0_ is the initial flux.

The fouling resistance were calculated according to the resistance-in-series model as displayed in Equation (5) [19]:

(5) $$R_{t}=\frac{TMP}{\mu J}=R_{m}+R_{r}+R_{ir}$$

where *R_t_*, *R_m_*, *R_r_* and *R_ir_* were the total membrane fouling resistance, intrinsic membrane resistance, hydraulic reversible and irreversible fouling resistance, which were calculated by the following equations:

(6) $$R_{m}=\frac{TMP}{\mu J_{0}}$$

(7) $$R_{t}=\frac{TMP}{\mu J_{2}}$$

(8) $$R_{r}=\frac{TMP}{\mu J_{2}}-\frac{TMP}{\mu J_{1}}$$

(9) $$R_{ir}=R_{t}-R_{m}-R_{r}$$

where *TMP* is trans-membrane pressure (Pa), *μ* is the dynamic viscosity for the feed water (Pa·*A* = s) and *J*_2_ is the permeate flux at the end of fouling filtration (m/s).

## 3. Results and Discussion

### 3.1. Characterization of Different Pal Nanoparticles

**Figure 2:**
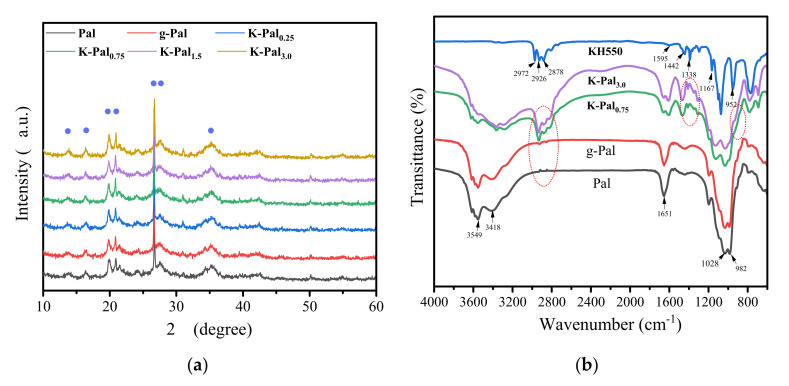
XRD patterns (**a**) and FTIR spectra (**b**) of different Pal nanoparticles and KH550.

The XRD and FTIR spectra of Pal, g-Pal and K-Pal nanoparticles were presented in Figure 2. As shown in Figure 2a, the peaks at 13.9, 16.4, 19.8 and 20.9° appeared, which were induced by the Si–O–Si crystalline layer of Pal [2,20]. The reflections at 2θ = 26.7 and 35.3° were inconsistent with the (2 3 1) and (0 0 2) planes of Pal [14,21]. After grinding or modification by KH550, it was found that the characteristic peaks of all the samples correspond basically, which suggested that the crystal structure of Pal was not destroyed [14]. From Figure 2b we can see the FTIR spectra Pal, g-Pal, K-Pal nanoparticles and KH550. For Pal, The absorbing peaks at 3549 and 3418 cm^−1^ are attributed to the to the stretching vibration of water molecules (i.e., zeolitic water and adsorbed water in the PAL crystal [16]. The band at 1651 cm^−1^ was mostly induced by the bending modes of the abovementioned water molecules groups. The absorbing peaks at 1028 and 982 cm^−1^ were attributued to the stretching vibration of Si–O bonds [15]. When the Pal nanoparticles were grinded to smaller size, it was seen that little difference was observed in the FTIR spectra. However, several typical absorbing peaks were investigated for KH550. The absorption peaks at 2926, 2878 and 1442 cm^−1^ were attributed to asymmetry stretching vibration, symmetry stretching vibration and deformation vibration of C–H, respectively. The absorption peaks at 1167, 2972 and 1595 cm^−1^ were corresponding to stretching vibration of C–C and stretching vibration and deformation vibration of N–H. The absorption peaks at 1338 and 952 cm^−1^ were C–Si–O [13]. After modification of Pal nanoparticles by KH550, it was obvious that some new peaks appeared in the K-Pal particle surface, and these new absorption peaks have been marked with red circle in the FTIR spectra and were same as the KH550, which demonstrated the successful graft to the surface of Pal through chemical reaction. Nevertheless, little difference of absorption peaks was detected in FTIR spectra between K-Pal_0.75_ and K-Pal_3.0_.

**Figure 3:**
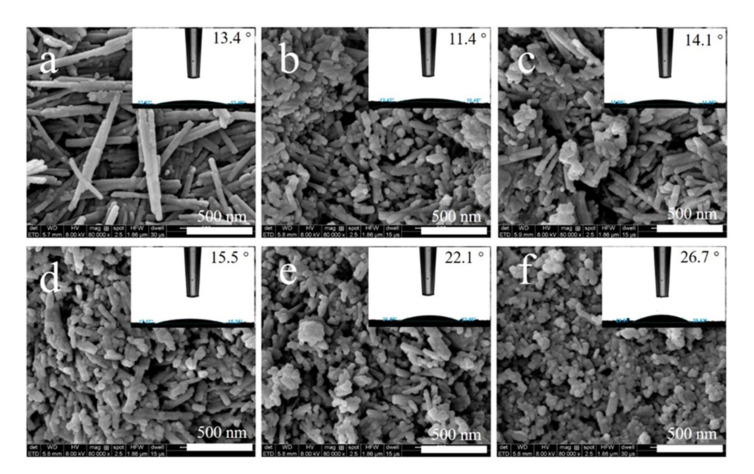
SEM images and contact angles (insert) of different Pal nanoparticles. (**a**) Pal, (**b**) g-Pal, (**c**) K-Pal_0.25_, (**d**) K-Pal_0.75_, (**e**) K-Pal_1.5_ and (**f**) K-Pal_3.0_.

Figure 3 displayed the SEM images and contact angles of Pal, g-Pal and K-Pal nanoparticles and the microstructure characterized by TEM was shown in Figure S1. As shown in Figure 3a, a typical rod-like morphology of Pal was observed with diameters of 30–50 nm and length of about 500–1000 nm in which the contact angle was 13.4° [1,6]. After grinding, the length was notably shortened to 100–150 nm, but not uniformly as displayed in Figure 3b. Besides, the contact angle showed a slight decrease, which was induced by the increased specific surface area and more hydroxyl groups appeared to the surface of Pal, and thus improved the hydrophilicity. When KH550 was grafted to the surface of Pal nanoparticles, the surface morphology and microstructure presented little changes. Nevertheless, when the mass ratio of KH550 and grinded Pal m/m was 3:1, it was found that some spherical particles appeared in in the surface of Pal as shown in Figure 3f, which may be induced by the hydrolysis of KH550 to the formation of silicon dioxide nanoparticles [22]. Furthermore, it was seen that the contact angle of K-Pal gradually increased from 14.1 to 26.7° with the increase in modification concentration of KH550. This phenomenon was mainly caused by the introduction of alkane chain on Pal surface and mades its surface less hydrophilic, despite the fact that KH550 also has a polar hydrophilic group (-NH_2_). Similar changes in trends were also investigated in KH550-modified Zeolite nanoparticles [14].

Particle size and zeta potential of Pal, g-Pal and K-Pal nanoparticles were summarized in Table 1. The Pal exhibited an average particle size about 876.8 nm, accompanied by a negative charge [2]. However, the poly-dispersion index of Pal was at a relatively high level, about 0.901, which indicated that the Pal particles were not uniform in particle length [23]. After grinding, the average particle size decreased to 513 nm, which was not in accordance with the particle size displayed in SEM and TEM images (100–150 nm). Though the poly-dispersion index showed an obvious decrease, the g-Pal still tended to aggregate, thus resulting in the increase in particle size characterized by DLS. When KH550 was grafted to the surface of Pal nanoparticles, the average particle size further decreased as well as the zeta potential. It was satisfactorily seen that K-Pal_0.75_ presented an average particle size about 312.6 nm with a relative lower poly-dispersion index, which means an improved uniform dispersion despite the aggregation of particles was not completely eliminated [24]. These results demonstrated the feasibility of the modification by KH550 in weaken the aggregation of Pal, so as to improve the dispersion in the preparation of TFN membranes.

Table 1 also summarized the settling rate of Pal, g-Pal and K-Pal in *n*-hexane solution. As obtained, after modification by KH550, the settling rate presented a sharp decrease from 8.67 × 10^−4^ cm/s to 1.22 × 10^−5^ cm/s. The Pal nanoparticles were abundant with hydroxyl groups, which made them a strongly polar substance and almost incompatible with n-hexane [14]. Conversely, the hydroxyl groups of K-Pal were effectively covered by the KH550 layer graft to the particle surface, and lots of organic groups were introduced to the Pal surface, and thus made its surface organophilic (hydrophilicity was weak), which was in accordance with the result of the contact angle. Therefore, compared with Pal, KH550-modified Pal had better compatibility and could uniformly stay longer in n-hexane, which was believed to improve the uniform dispersion of particles in the PA layer and reduce the introduction of a large number of defects, thus leading to a higher performance of the TFN membranes. Considering the average particle size associated with the low poly-dispersion index and settling rate, the mass ratio of KH550 and g-Pal m/m at 3:4 (K-Pal_0.75_) was selected to fabricate TFN membranes.

### 3.2. FTIR, SEM and AFM Characterization of RO Membranes

**Figure 4:**
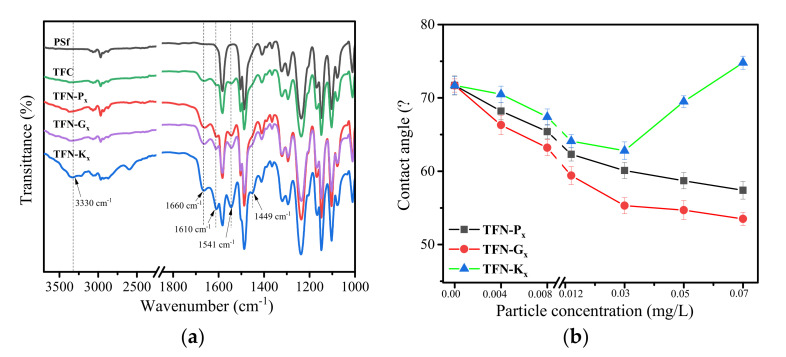
FTIR spectra (**a**) and contact angle (**b**) of different membranes.

FTIR spectra of PSf, TFC, TFN-P_x_, TFN-G_x_ and TFN-K_x_ membranes were shown in Figure 4a. For PSf, the characteristic peaks appeared at 1150, 1242, 1488 and 1585 cm^−1^, which belonged to the asymmetric O=S=O stretching vibrations, asymmetric C–O–C stretching, symmetric O=S=O stretching and aromatic bands stretching [25]. After interfacial polymerization occurred on the surface of PSf, the typical peaks of the PA layer at 1541, 1610 and 1660 cm^−1^ were both detected in the TFC and TFN membranes, which was related to the amide II N–H bending and torsional motion, hydrogen-bonded C=O stretching vibration and amide I C=O stretching vibrations, respectively [26]. Besides, it was seen that the FTIR spectra of TFN-P_x_ and TFN-G_x_ membranes showed little difference, but both of them exhibited an intensified broad band at 3330 cm^−1^ compared to TFC membranes, which was ascribed to the hydroxyl groups of Pal and g-Pal nanorods [27]. Moreover, the absorption peak of KH550 at 1381 cm^−1^ (Figure 2) happened to blue shift and the new peak at 1449 cm^−1^ was attributed to the deformation vibration of C–H provided by KH550.

The contact angles of TFC and TFN membranes were shown in Figure 4b. For TFN-P_x_ and TFN-G_x_ membranes, with the increased loading concentration of Pal and g-Pal nanoparticles in the PA layer, the contact angle presented a declined trend, resulting from the existence of hydrophilic groups on the Pal nanoparticles [28]. Since the g-Pal nanoparticles was more hydrophilic that has been proven by the contact angle of powder in Figure 3, more hydrophilic groups existed on the particle surface, thus the TFN-G_x_ membranes exhibited a lower contact angle value and indicated higher hydrophilicity than the TFN-P_x_ membranes. However, the contact angle of TFN-K_x_ membranes showed a different trend. For TFN-K_x_ membranes, when the loading concentration of K-Pal was below 0.03 mg/L, the contact angle remained in a decreased state with the increase in loading concentration. Though the hydroxyl groups in the Pal surface was covered by the KH550 layer, the existence of NH_2_ in KH550 also played a positive role in enhancing the hydrophilicity of the TFN-K_x_ membranes. However, when the loading concentration further increased, the contact angle of TFN-K_x_ membranes gradually gained increment. This might be attributed to the sharp increase in membrane roughness as shown in Figure S2, which hindered the wettability of the membranes. Besides, the introduced alkane chain of overdosed K-Pal may be another reason for the contact angle increment despite the fact that KH550 also has polar hydrophilic group (–NH_2_) [21,29].

**Figure 5:**
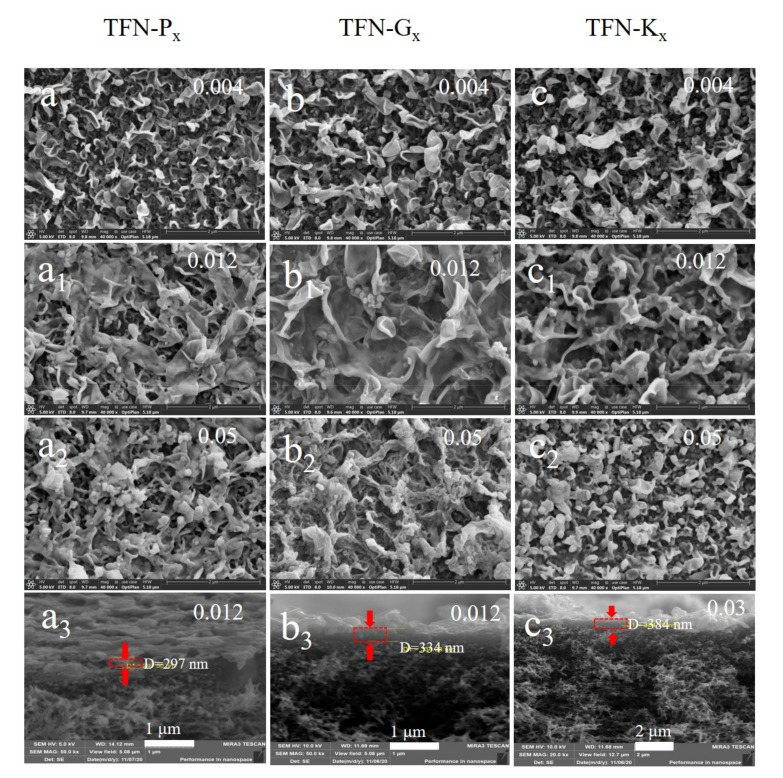
Surface morphology and cross-section of TFN-P_x_ (**a**–**a_3_**), TFN-G_x_ (**b**–**b_3_**) and TFN-K_x_ (**c**–**c_3_**) membranes.

The surface morphology of the TFN membranes under different loading concentration of Pal, g-Pal and K-Pal particles were presented in Figure 5, and the images of other prepared membranes were displayed in Figure S2. Apparently, a typical “ridge-and-valley” structure occurred on both TFC and TFN membranes, suggesting the successful formation of PA layer on the PSf substrate [25,26]. When Pal, g-pal and K-Pal were added in organic phase, the “ridge-and-valley” structure changed to larger “leaf-like” morphological structures of all the TFN membranes, and the thickness of the PA layer showed a slight increase in thickness compared to TFC membranes as seen in the cross-section images (Figure S2). This phenomenon was induced by enhanced miscibility of aqueous and organic phases upon the addition of hydrophilic nanoparticles, thus the expanded interfacial polymerization reaction zone that has been detailed explained by previous studies [30,31]. For TFN-P_x_ and TFN-G_x_ membranes, when the loading concentration of particles was 0.012 wt%, slight aggregation of Pal or g-Pal was observed in the membrane surface and further increment of loading concentration resulted in more severe aggregation. By contrast, the TFN-K_x_ showed a uniform dispersion of K-Pal nanoparticles till a slight aggregation occurred when the loading concentration at 0.05 wt%. This satisfied result was mainly caused by the graft of KH550 to the Pal surface and enhanced the compatibility between K-Pal nanoparticles and TMC organic phase, which was in accordance with the settling analysis.

**Figure 6:**
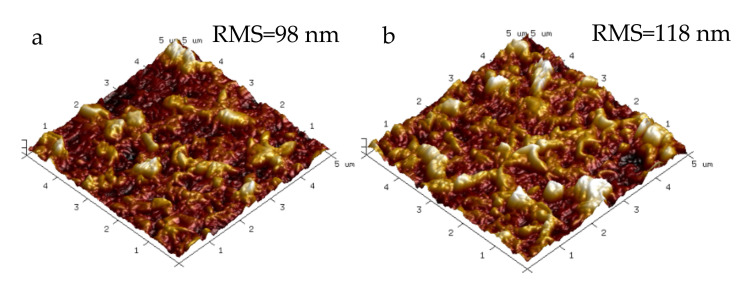

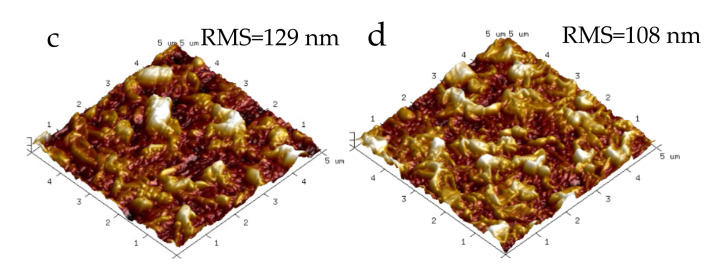
AFM images of (**a**) TFC, (**b**) TFN-P_0.012_, (**c**) TFN-G_0.012_ and (**d**) TFN-K_0.03_ membranes.

The surface roughness of the RO membranes was investigated by AFM, and the 3D images were displayed in Figure 6. Compared to TFC membrane, the surface roughness of the TFN membranes were all increased, which was mainly caused by the larger “leaf-like” morphological structures induced by incorporation of Pal, g-Pal and K-Pal nanoparticles. The increased surface roughness may lead to more contact area with water molecules, and hereby contributed to enhancing the water flux [2]. It is noticed that the surface roughness of TFN-P_0.012_ and TFN-G_0.012_ membranes was higher than TFN-K_0.03_ membranes, which was induced by the slight aggregation of nanofillers in accordance with the SEM images.

### 3.3. Separation Performance of RO Membranes

**Figure 7:**
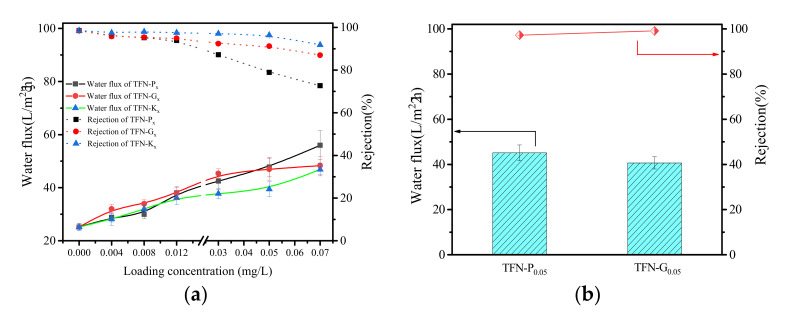

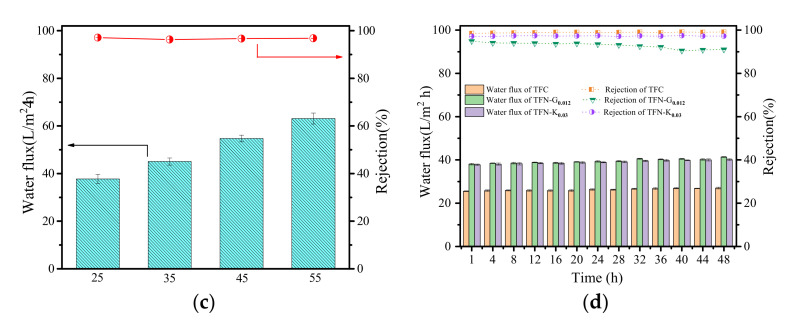
Water flux and NaCl rejection of the TFN membranes with different loading concentration of Pal, g-Pal and K-Pal (**a**), water flux and MgCl_2_ rejection of TFN-P_0.05_ and TFN-G_0.05_ membranes (**b**), water flux and NaCl rejection of TFN-K_0.03_ under different temperature (**c**) and water flux and NaCl rejection of TFC, TFN-G_0.012_ and TFN-K_0.03_ membranes under long-term RO test (**d**).

Figure 7 reveals the water flux and salt rejection trends of the nanocomposite membranes prepared with Pal, g-Pal and K-Pal nanoparticles under different conditions, respectively. As obtained from Figure 7a, the TFC membrane exhibited a water flux of 25.17 L/m^2^·h with 98.5% NaCl rejection. All the three types of TFN membranes showed an increment in water flux after incorporation of Pal, g-Pal, and K-Pal in the rejection layer and the higher the concentration, the greater the flux obtained. As discussed above, addition of Pal, g-Pal, and K-Pal was benefited to improve the membrane roughness and hydrophilicity, which enlarged the contact area with water molecules and facilitated the water solubilization and diffusion through the rejection layer [32]. Besides, the tubular structure of Pal with a cross-sectional area 0.37 × 0.63 nm^2^ could provide more high-speed nanochannels for water transport [33]. It was notable that the salt rejection presented different change trends of the three types of TFN membranes. For TFN-P_x_ and TFN-G_x_ membranes, the salt rejection showed a visible drop even at a low loading concentration. The slight aggregation of Pal and g-Pal at low loading concentration may bring nanocorridors between nanofillers and polyamide matrix in the rejection layer, resulting in the declination of NaCl rejection [2]. In addition, the larger particle size may hinder the IP process, and destroy the integrity of the PA layer [34]. When the loading concentration exceeded 0.012 wt%, the decline was more obvious, resulting from the severe aggregation, and more defects were generated on the membrane surface as observed in SEM images. Furthermore, it was emphasized that the extent of the reduction in NaCl rejection for TFN-G_x_ membranes was much lower than that for TFN-P_x_ membranes, which can be explained by the decreased particle size that tended to be covered more in the PA layer [35].

Different from the TFN-P_x_ and TFN-G_x_ membranes, the decline of NaCl rejection of TFN-K_x_ membrane appared till the loading concentration increased to 0.05 wt%. The graft of KH550 to the Pal may weaken the aggregation and enhance the uniform dispersion in PA layer, and the existence of NH_2_ in K-Pal surface may react with TMC molecules and improve the compatibility between nanoparticles and PA layer, thus leading to less defects and the better separation performance. However, when the loading concentration of K-Pal further increased to 0.07 wt%, the membrane also displayed a decrease in NaCl rejection. It must be emphasized that the chemical modification was only the way to weaken the aggregation of inorganic nanoparticles and increase the loading concentration in the membrane-selective layer without affecting the membrane performance, but not to elimate the aggregation. Thus, it was essential to define the optimal loading concentration by using inorganic nanoparticles as nanofillers rather than the more the better. Figure 7b depicted the separation performance of TFN-P_0.05_ and TFN-G_0.05_ membranes by using 2 g/L MgCl_2_ as feed solution. Though the two membranes exhibited bad rejection to NaCl at 0.05 wt% loading concentration caused by the severe aggregation of Pal and g-Pal, the rejection to MgCl_2_ reached 97.1% for TFN-P_0.05_ and 99.2% for TFN-G_0.05_ membranes with excellent enhanced water flux, respectively. Since the Mg^2+^ has a bigger hydrated ionic radius, it was harder to be transparent across the membrane despite some defects occurring in the PA layer. Simultaneously, the counter-ions (Cl^−^) were rejected by the Donnan effect, which contributed to the higher rejection of MgCl_2_ [18,36].

Several studies have reported that the polymer membrane is very sensitive to the changes in the feed temperature [37]. Figure 7c showed the separation performance of TFN-K_0.03_ membranes by adjusting the temperature of the feed solution. It was obvious that up to a 60% enhancement in water flux when the feed temperature was adjusted from 25 to 55 °C, which may be caused by the changes in the physical properties of the polymeric membrane such as the pore size or possibly the diffusivity of water molecules in the membrane [38]. Besides, the increase in temperature may effectively decrease the degree of concentration polarization (CP) resistance, which also contributed to the high transmembrane flux [39]. It was satisfied that the salt rejection remained unchanged under different temperatures of the feed solution, suggesting the stability of K-Pal nanoparticles in the PA layer which was ascribe to the successful graft of KH550 in the particle surface.

In order to investigate the membrane stability, a 48 h RO test was conducted and the separation performance was shown in Figure 7d. As obtained, the water flux and rejection of TFC and TFN-K_0.03_ membranes exhibited slight increases during 48 h filtration, which indicated that robust rejection layers were formed and K-Pal nanoparticles stably existed in the rejection layer. The slight enhancement of water flux was mostly caused by the changes in the physical properties of the polymeric membrane discussed above. On the contrary, the rejection of TFN-G_0.012_ gradually decreased, especially after 32 h operation. Compared to g-Pal, the K-Pal nanoparticles could uniformly disperse in the PA layer and the reaction between NH_2_ in K-Pal surface and TMC molecules could make the particle firmly fixed in the rejection layer. While the G-Pal nanoparticles were kept in the PA layer only by physical package, it could easily induce membrane defects under the constant rush of water at high pressure, thus leading to the deterioration of salt rejection [7]. Furthermore, the effect of operating pressure on the membrane separation performance was also studied, which was displayed in Figure S3.

Table 2 compares the RO performance with different porous nanomaterials. The TFN-Kx membrane in this work exhibited a better flux increase ratio and rejection than some other nanoparticles, though it was not the best. These results demonstrated the positive effect of modification of Pal by KH550, which could effectively improve the membrane performance.

### 3.4. Antifouling Capacity

**Figure 8:**
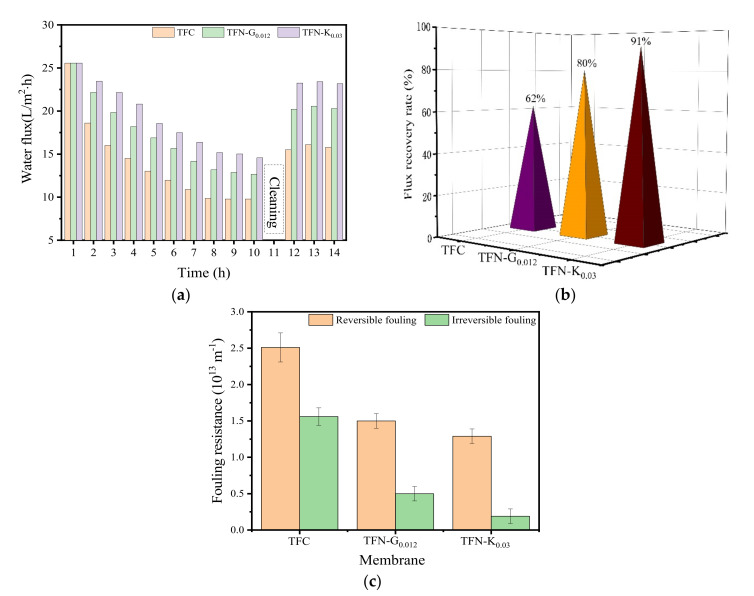
The antifouling capacity of the TFC, TFN-G_0.012_ and TFN-K_0.03_ membranes. (**a**) Time-dependent flux for HA; (**b**) FRR of TFC, TFN-G_0.012_ and TFN-K_0.03_ membranes and (**c**) fouling reversibility.

Membrane fouling is an important and inevitable phenomenon that impairs the performance of membranes during RO application [42]. In the current study, HA was used as model foulant to conduct the fouling tests so as to evaluate the effect of particle size and modification of KH550 on the antifouling ability of the TFN membranes, and the results were shown in Figure 8. As shown in Figure 8a, the same initial flux of 25.5 L/m^2^·h was first adjusted for TFC, TFN-G_0.012_ and TFN-K_0.03_ membranes to obtain an identical transverse hydrodynamic force for each membrane [2]. For TFC membranes, it suffered a sharp decline and reached a relative steady state after about 8 h when HA was added in the feed solution, and the final water flux was only 38% of the initial flux. This phenomenon suggested that membrane fouling caused by HA was fast but could reach a balance in a certain time. As a kind of hydrophilic polymer, HA could accumulate at the membrane surface and add the hydraulic resistance, thus aggravating water flux decline [43]. After cleaning, the water flux merely recovered to 62%, which was caused by the inevitable membrane fouling. Since RO is a pressure-driven process, the HA molecule accumulated on the membrane surface would be compacted during the filtration process, the boundary layer was hardly flushed away under high cross-flow rate and the membrane pores were completely covered, leading to the great resistance to the water molecules passing across the membrane, resulting in the low recovery of the water flux [44].

Compared to TFC, TFN-G_0.012_ and TFN-K_0.03_ membranes showed a relatively slow decline trend and lower pollution level. The flux recovery was 80% for the TFN-G_0.012_ membrane and 91% for the TFN-K_0.03_ membrane. Since the initial flux was operated to the same by adjusting the pressure, the operating pressure of the TFN-G_0.012_ and TFN-K_0.03_ membrane was much lower than TFC, thus the boundary layer was more loose than that on the TFC membrane surface and more easily flushed away under a high cross-flow rate, thereby enhancing the flux recovery rate [5]. Besides, the enhanced hydrophilicity of the TFN-G_0.012_ and TFN-K_0.03_ membrane surface may have also contributed to the higher final flux and flux recovery rate. Furthermore, it was noticed that TFN-K_0.03_ membranes exhibited higher flux recovery rate than TFN-G_0.012_ membranes, and the lower surface roughness of TFN-K_0.03_ membranes may be the most plausible explanation [45].

The fouling reversibility displayed in Figure 8c further suggested the membrane fouling phenomenon. TFC membranes suffered the most severe reversible and irreversible fouling, with fouling resistances of 2.51 × 10^13^ and 1.55 × 10^13^ m^−^^1^, respectively. The high level of irreversible fouling was mainly caused caused by the pore blocking of the TFC membranes, and it was hardly removed by the simple hydraulic backwash, thus inducing the severe flux decline and low FRR. On the contrary, the TFN-K_0.03_ membranes displayed the lowest reversible and irreversible fouling, with the fouling resistances of 1.29 × 10^13^ and 0.19 × 10^13^ m^−1^. The result of fouling reversibility showed that reversible fouling played a dominant role in TFN-K_0.03_ membranes [19]. Furthermore, it is noticed that the irreversible fouling of TFN-K_0.03_ membranes was much lower than that of TFN-G_0.012_ membranes, which was attributed to the well dispersion of K-Pal nanoparticles and lower surface roughness of the membrane surface. Less defects, enhanced hydrophilicity and lower surface roughness of TFN-K_0.03_ membranes made a great contribution to the higher FRR and lower irreversible fouling than both TFN-G_0.012_ and TFC membranes.

## 4. Conclusions

In the current study, Pal, g-Pal and K-Pal nanoparticles were incorporated into the PA layer to evalute the effect of particle size, loading content and KH550 modification of Pal on the water flux, salt rejection, stability and antifouling capacity of the prepared TFN membranes. The results showed that when the mass ratio of KH550 and g-Pal was 3:4, the K-Pal possessed the lowest particle size (312 nm) and settling rate (1.22 × 10^−5^ cm/s). The RO separation performance suggested that the membrane with K-Pal performed the best in enhancing the water flux and maintaining the salt rejection. When the loading concentration of K-Pal was 0.03 wt%, the water flux increased from 25 to 38 L/m^2^·h and the salt rejection remained almost unchanged. On the contrary, the rejection of the membrane with raw Pal and g-Pal exhibited declination even at a low incorporating concentration. However, it was noticed that these two kinds of membranes showed excellent rejection to MgCl_2_: 97.1% for TFN-P_0.05_ and 99.2% for TFN-G_0.05_ membranes, at the concentration of 0.05 wt% with excellent water flux. Finally, the membrane with K-Pal exhibited better stability, fouling resistance and FRR than the TFN-G_x_ and TFC membranes and the FRR even exceeded 90% of the TFN-K_0.03_ membranes, which contributed to the improved hydrophilicity and lower roughness of the membrane surface than TFN-G_0.012_ membranes.

## Figures and Tables

**Table 1:** Physical properties of Pal, g-Pal and K-Pal nanoparticles.

Samples	Zeta Potential (mV)	Mean Diameter (nm)	Poly-Dispersion Index (PDI)	Settling Rate (cm/s)
Pal	−11.7 ± 0.6	876.8 ± 122.8	0.901 ± 0.134	8.67 × 10^−4^
g-Pal	−12.0 ± 0.4	513.0 ± 14.5	0.412 ± 0.126	4.81 × 10^−4^
K-Pal_0.25_	−12.8 ± 0.4	436.0 ± 12.2	0.373 ± 0.155	7.47 × 10^−5^
K-Pal_0.75_	−13.2 ± 0.8	312.6 ± 6.2	0.113 ± 0.058	1.22 × 10^−5^
K-Pal_1.5_	−13.8 ± 0.2	385.3 ± 15.0	0.419 ± 0.119	1.56 × 10^−5^
K-Pal_3.0_	−14.5 ± 0.7	411.7 ± 9.0	0.155 ± 0.074	2.08 × 10^−5^

**Table 2:** Comparisons of separation performances with differert porous nanomaterials.

Nanoparticles	Loading at Best Performance ^a^	Pressure (bar)	PWP (L/m^2^·h·bar) ^b^	NaCl Rejection (%) ^b^
MCM-48 [26] nanoparticles	0.10 wt/v% (O)	16.0	1.50 → 2.18	97.0 → 97.0
Halloysite nanotubes [3]	0.05 wt/v% (O)	15.0	1.27 → 2.41	97.2 → 95.6
ZIF-8 [40]	0.15 wt% (O)	20.0	1.72 → 2.61	98.2 → 98.6
Layered double hydroxides [41]	0.2 wt% (O)	20.0	1.49 → 2.75	98.5 → 99.05
Pal/TiO_2_ [2]	75 mg/L (A)	16.0	1.53 → 2.13	98.2 → 98.0
Pal/Ag [6]	7.5 mg/L (A)	16.0	1.50 → 2.49	98.5 → 98.3
Pal-KH550 (this work)	0.03 wt% (O)	16.0	1.57 → 2.38	98.5 → 98.0

^a^ (A) means the nanoparticles are in the water phase, while (O) means in the organic phase. ^b^ The left numbers are the performances of the control membranes, while the right ones are the performances corresponding to the optimal loadings.

## Data Availability

Not applicable.

## Supplementary Materials

The following are available online at https://www.mdpi.com/article/10.3390/membranes11110889/s1, Figure S1. TEM images of different Pal nanoparticles, Figure S2. SEM images of TFC, Figure S3. Water flux and NaCl rejection of TFC.
