# Supplementary material for: Organo-Functionalization: An Effective Method in Enhancing the Separation and Antifouling Performance of Thin-Film Nanocomposite Membranes by Improving the Uniform Dispersion of Palygorskite Nanoparticles
Source: Membranes (Basel). 2021 Nov 19;11(11):889. doi: 10.3390/membranes11110889 (PMC8624518; doi:10.3390/membranes11110889)
Supplement: Supplementary file 1 [file membranes-11-00889-s001.zip › membranes-1467258-supplementary.pdf]

Organo-Functionalization: An Effective Method in Enhancing the Separation and Antifouling Performance of Thin-Film Nanocomposite Membranes by Improving the Uniform Dispersion of Palygorskite Nanoparticles

Liu Yang [1], Qianwen Zhang [2], Qikun Wang [1], Wande Ding[1,3,*], Kefeng Zhang [1]

[1] School of Municipal and Environmental Engineering, Shandong Jianzhu University, Jinan 250101, China; 15665888192@163.com (L.Y.); qikunwang12@gmail.com (Q.W.); kfz@sdjzu.edu.cn (K.Z.)

[2] School of Water Resources & Environment, China University of Geosciences, Beijing 100083, China; zhangqw@caep.org.cn

[3] Shandong Shuifa Environmental Technology Co., Ltd., Jining 272000, China

* Correspondence: dingwande18@sdjzu.edu.cn

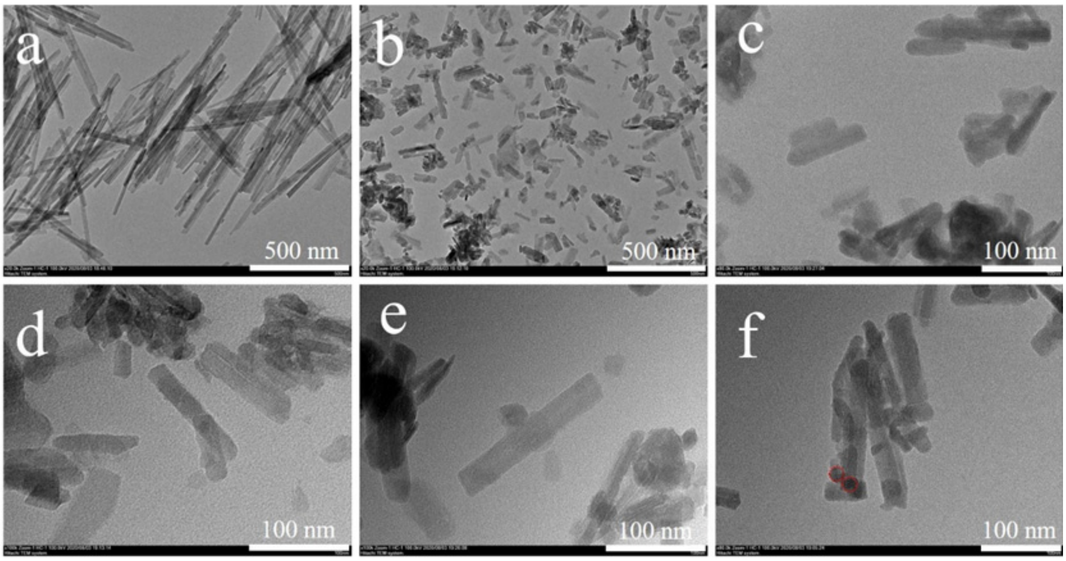

Figure S1. TEM images of different Pal nanoparticles. (a) Pal, (b) g-Pal, (c) K-Pal$_{0.25}$, (d) K-Pal$_{0.75}$, (e) K-Pal$_{1.5}$ and (f) K-Pal$_{3.0}$.

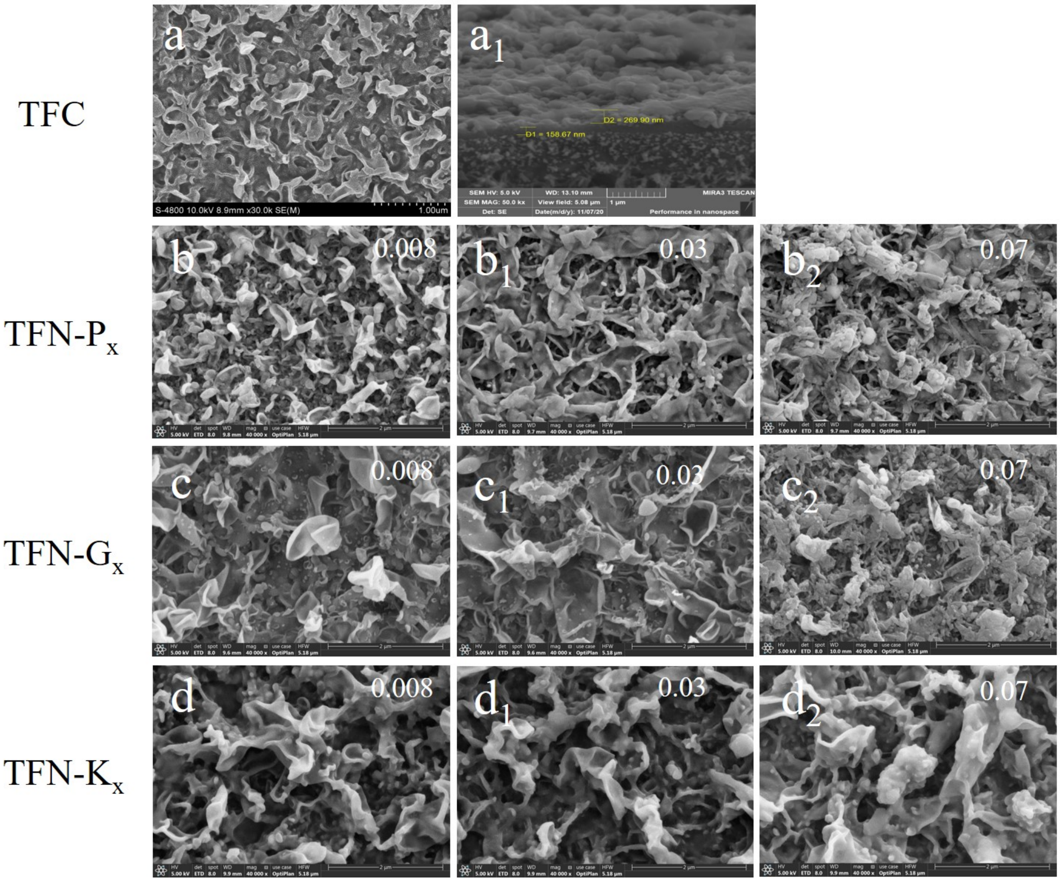

Figure S2. SEM images of TFC (a, a_1), TFN-P_x (b-b_2), TFN-G_x (c-c_2) and TFN-K_x (d-d_2) membranes.

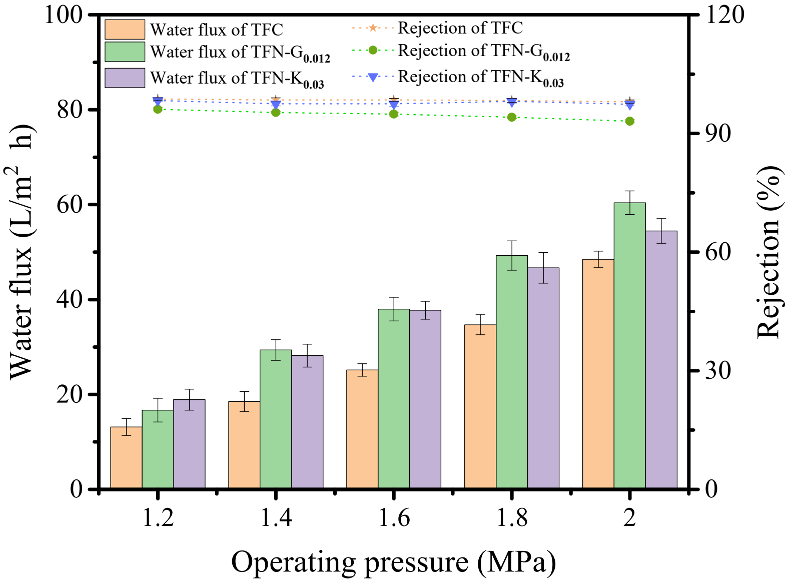

Figure S3. Water flux and NaCl rejection of TFC, TFN-$G_{0.012}$ and TFN-$K_{0.03}$ membranes under different operation pressure.
